# Molecular dynamics study on structural and atomic evolution between Au and Ni nanoparticles through coalescence

**DOI:** 10.1038/s41598-021-94822-0

**Published:** 2021-07-29

**Authors:** Bangquan Li, Jing Li, Xiaoqiang Su, Yimin Cui

**Affiliations:** 1 School of Physics and Electronics, Shanxi Datong University, Datong, 037009 China; 2 Institute of Photoelectronic Thin Film Devices and Technology, Nankai University, Tianjin, 300350 China; 3 School of Physics, Beihang University, Beijing, 100191 China

**Keywords:** Nanoparticles, Molecular dynamics

## Abstract

Motivated by the structure evolution experiments of Janus NiAu nanoparticles (NPs), we present a detailed study on the thermodynamic evolution of Ni and Au NPs with different ratios of Au and Ni through the molecular dynamics (MD) simulations. It is found that, for fixed Ni particle size (5.8 nm in diameter), the energy variation with the increasing temperature is related to the Au sizes (1.5–9.6 nm in diameter), due to the diverse atomic segregation modes. For a small Au particle, due to lattice induction, the structure will change from order to disorder and then to order. The interface defects of the merging NPs could be automatically eliminated by coalescence processes. The change in energy as the temperature increases is similar to that of monometallic NPs. For larger Au particles, the irregular variation of energy occurs and the atomic energy experience one or two reductions at least with the increase of the temperature. The segregation of Au atoms to the surface of Ni particle is dominant during the continuous heating process. The coalescence processes of Au atoms strongly determine the final morphology of the particles. Dumbbell-like, Janus and eccentric core–shell spherical structures could be obtained during the heating process. Our results will provide an effective approach to the design of novel materials with specific properties through thermal control.

## Introduction

It is well known that the physical and chemical properties of metallic nanomaterials depend strongly on their size and morphology^1–3^. Heterogeneous bimetallic nanoparticles (NPs) consisting of magnetic and noble metals possess enhanced magnetic, optical, and catalytic performance compared to the corresponding monometallic NPs^4,5^. In addition, the designing of the spatial arrangement and interfacial structure of NPs could further promote performance optimization and reveal structure–property relationship. Coalescence is an important growth mode in particle formation processes^6,7^. Coalescence of NPs and the evolution of their shapes have been widely investigated in recent years. For instance, the coalescence of decahedral gold NPs was investigated with the in-situ TEM technique, and the faceted structure of NPs was found to play a crucial role in agglomeration and coalescence^8^. As soft particles, when the silver NPs came into contact and underwent a spontaneous coalescence process, they could form the conductive films^9^. The metastable structures of bimetallic Ag–Cu NPs, such as Janus or Ag@Cu core/shell, were produced after coalescence below the melting point^10^. In-situ TEM images showed that Ostwald ripening process was found to be more prevalent at higher temperature during the sintering process of Ag and Pt NPs^11^. Molecular dynamics (MD) simulations revealed that coalescence of iron nanoclusters occurred at a temperature lower than the melting point of the clusters, and the difference in temperatures between coalescence and melting increased as the decreasing of cluster size^12^. The collision and coalescence of gold clusters indicated that the morphology and structure of the final particle were determined strongly by the coalescence processes^13^. The energy decrease was mainly caused by the reduction of surface energy occurred with coalescence of Ag and Pd clusters. The Pd atoms preferred staying inside the cluster with Ag coming to the surface^14^. The sintering procedure of FeNi NPs resulted in phase-segregated particles that are comprised of Fe-enriched surface and Ni-enriched core^15^. In our previous work, the Au-Ni Janus NPs were synthesized in a mild chemical solution system. The structure evolution behaviors were studied using an in-situ Cs-corrected STEM method at atomic scale^16^.

The understanding of coalescence processes may have lots of potential applications in finding optimal approaches to control the size and shape of the particles. However, the structure evolution derived mainly from surface atoms, which only reveals the composition of the surface layer, cannot identify the atom evolution of the inner core or interfaces. On the other hand, magnetic metal Ni have smaller atom size and higher surface energy compared to the noble metal Au, rendering the immiscibility under equilibrium conditions with a large lattice mismatch (e.g., ~ 15.7% between Ni (3.524 Å) and Au (4.079 Å) with a face centered cubic (FCC) structure)^17^. Benefitting from synergistic effect, it is reasonable to design and control Au-Ni structures by engineering the size, composition and atomic configuration in order to acquire distinctive properties.

The MD simulations with classical potentials method is known to be a tool of great reliability in the study of structural evolution and dynamic information of NPs^18–20^. In this work, we carried out a comparative study of segregation characteristics of coalescence process between Au and Ni NPs by MD simulations. Our results will provide both an effective approach to design novel materials with specific properties by thermal controlling and a perspective on the application of bimetallic NPs in high temperature environments.

## Methods

The embedded atom method (EAM) was adopted to describe the interatomic interaction of Au-Ni. In the EAM force field^21^, the potential parameters were optimized according to basic material properties such as lattice constants, elastic constants, bulk moduli and vacancy formation energies, which provide a reasonable approximation to the interactions between different metal elements. In a bimetallic system, the total potential has been successfully applied to the Ni-Au system at the nanometer scale^22,23^.

Our MD simulations are performed with large-scale atomic/molecular massively parallel simulator (LAMMPS) developed by Sandia National Laboratories^24^. The initial and output graphics have been created by VMD 1.9.2 software package (http://www.ks.uiuc.edu/Research/vmd/)^25^. The MD simulations were performed in the canonical NVT (fixed number of particles, volume and temperature) ensemble using the Nose–Hoover thermostat for maintaining the temperature. Typically, the equations of motion were integrated using the velocity-Verlet integrator with a time step of 0.001 ps.

**Figure 1:**
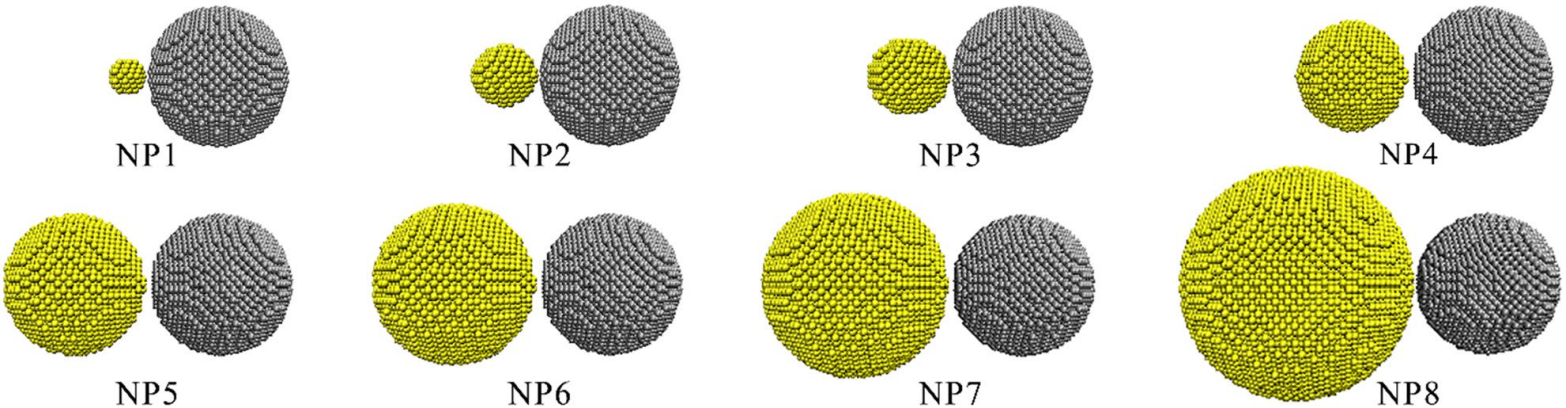
Initial configurations of Ni-Au NPs structures with different atomic ratio, Au and Ni atoms are colored in yellow and gray, respectively.

As shown in Fig. 1, spherical gold and nickel NPs are extracted from a bulk FCC crystal with the [100], [010] and [001] crystallographic directions along the *x*-, *y*- and *z*-axes, respectively. A perfect FCC lattice structure was constructed as the initial model with the corresponding lattice constants being 3.524 Å and 4.079 Å for Ni and Au, respectively. As initial simulated objects, Au-Ni structures contained variable Au atoms (from 96 to 27,311) and 9092 Ni atoms. That is, for each pair, Ni nanoparticle has the same diameter of 5.8 nm and the diameter of Au nanoparticle is varied, ranging from 1.5 to 9.6 nm (shown in the Table S1). The corresponding eight different concentrations are considered to be Au_0.01_Ni_0.99_ (NP1), Au_0.05_Ni_0.95_ (NP2), Au_0.1_Ni_0.9_ (NP3), Au_0.25_Ni_0.75_ (NP4), Au_0.4_Ni_0.6_ (NP5), Au_0.5_Ni_0.5_ (NP6), Au_0.6_Ni_0.4_ (NP7) and Au_0.75_Ni_0.25_ (NP8). The interaction of nanoparticles is affected by different crystallographic orientations^26^. In order to compare different models, Au and Ni NPs are placed with one another (along the x axis), at a distance equal to the length of Ni lattice constant.

Prior to starting the MD simulations, the NPs system was relaxed thoroughly at 0.1 K until the system reaches a steady state, then these NPs were subjected to a continuous heating with the heating rate of 0.2 K/ps in each simulation when the temperature increased from 0.1 to 2100 K, and lasting 300 ps relaxation at 2100 K, which is far above the melting point of bulk Au and Ni, so as to melt the whole NPs.

## Simulation Results and discussion

**Figure 2:**
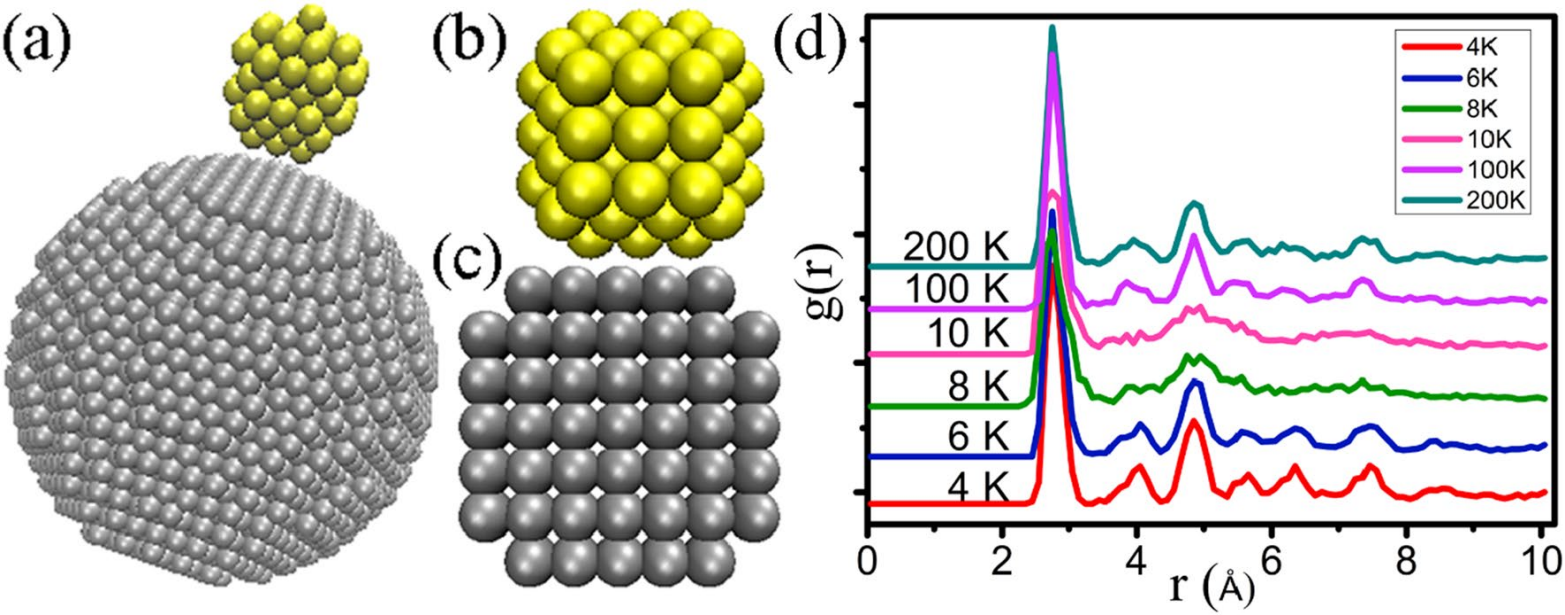
(**a**) The configurations of the simulated NP1 after a relaxation (**b**) (100) face of Au atoms (**c**) (100) face of Ni atoms (**d**) PDF of Au atoms in various temperatures.

By slightly heating the configurations, we can get insight into the shape changes and melting processes of such congeries. In a heating process, the two NPs are drawn together quickly by the excess surface energy. Subsequently, two NPs constantly change their positions, looking for state in space in order to find a lowest energy configuration^27^. Figure 2 shows snapshots of the NP1 during the simulation process. The two NPs are initially separated without heating applied yet (in Fig. 2a), and the atoms arrangements of the (100) of Au and Ni are shown in Fig. 2b,c. Before the coalescence of the Au atoms of NP1, the particles undergo the shape convulsion which has been termed “quasimelting”, and then coalesce into the large one at a highly accelerated rate compared with its prior migration. The approach and rotation of the two NPs can be clearly seen in the Supporting Information, Video S1. The small Au particle start heating, Au atoms and Ni atoms of (100) face hold atomic arrangement FCC structure (Fig. 2b,c), During heating process, the pair distribution function (PDF) of Au atoms and Ni atoms are also employed to monitor particle structure evolutions during the simulation. As shown in Fig. 2d, only first-nearest neighbor peaks of Au atomic arrangement can be seen in the PDF of the NP1 at 10 K, and the crystalline peaks disappear, while the FCC structure is restored at 100 K. When the temperature is less than 100 K, Au NPs and Ni NPs did not contact. The Au NPs transform or rotate in space in order to find a low-energy configuration (Au NPs are not the FCC) and then Au and Ni NPs are drawn together quickly by the excess surface energy, which is similar to the experiment was observed^28^. Subsequently, when the nanoparticle is small, its atoms are incorporated into the structure until almost every atom of the nanoparticle has rearranged over the (100) surface of the Ni NPs. We have studied the same size of the single element Au without Ni conditions, in which the Au structure has not changed in the same temperature. That is to say, the coalesced clusters with larger Ni NPs form an FCC structure. Structural transformation was induced by NPs containing greater numbers of atoms^29^.

**Figure 3:**
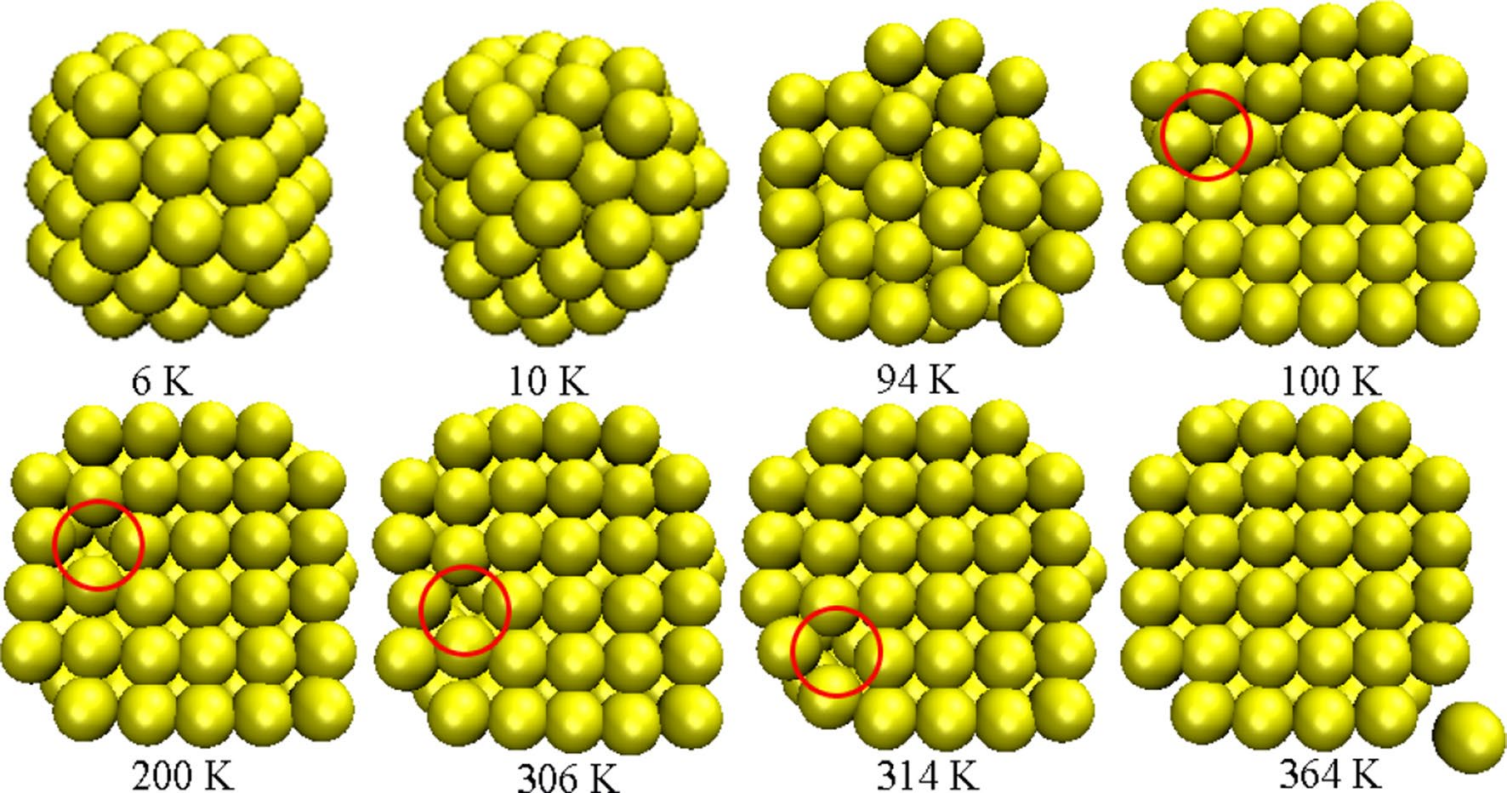
Snapshots of cross sections of Au atoms in the Au-Ni bimetallic NPs taken at eight representative temperatures (unchanged of (100) face of Ni atoms, as shown in the Fig. 2c).

Au atoms are induced by Ni atoms lattice, and the cross section of Au atoms can also illustrate this point. As shown in the Fig. 3, when the temperature is 6 K, the Au atoms of NP1 are arranged in an orderly manner. When the temperature is 10 K, the structure is deformed, and at a temperature of 100 K, Au atoms restore the ordered structure. In temperature range from 100 to 364 K, the atoms are slightly disturbed near the equilibrium position, and the nanoparticle structure is almost unchanged due to a strong coupling effect between Au atoms and Ni atoms. We also found that in the process of merging, the interface defects disappeared in Fig. 3. When the temperature is 94 K, Au particle and Ni particle start to contact, and soon Au atoms in accordance with Ni (100) arrangement. The defects were observed during the process. As the temperature increases, the defects are gradually shifted and eliminated. These results allowed us to identify the remaining defects are originated from the coalescence phenomenon mentioned previously, which can be eliminated by means of energy input into the system. The eliminate process can be clearly seen in the Supporting Information, Video S2. During the coalescence process, the Au atoms showed “flow” characteristics and “flowed” toward the neighboring Au NP because of the atomic interaction forces^30^. On the other hand, the NPs tend toward a more stable structure, displacing defects and releasing internal stresses^31,32^. These findings suggested that thermal energy could be applied to reduce the number of coalescence defects, which might cause a poor performance. It is also found that, with the increasing of Au atoms, Au atoms and Ni atoms interface are still ordered. We believe that no matter how many the number of Au atoms, Ni content will induce Au in an orderly arrangement over the (100) surface due to Au atoms more fluidity.

**Figure 4:**
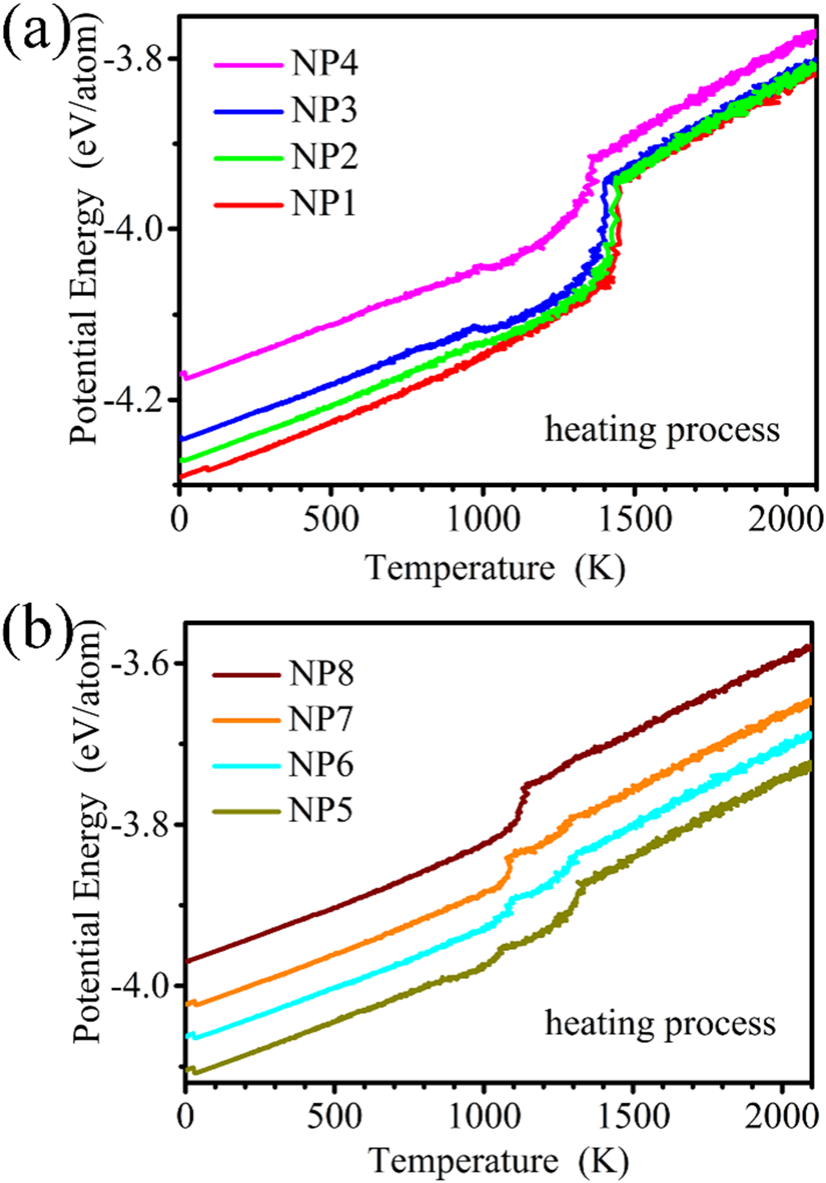
Variation of potential energy as function of temperature for the NPs heating process (**a**) NP1, NP2, NP3, NP4 (**b**) NP5, NP6, NP7, NP8.

To analyze the stability and structural transformation of Ni-Au NPs, the caloric curves of potential energy variation on temperature is illustrated in Fig. 4. The caloric curve of metallic nanomaterials is very different from that of bulk materials due to the decrease in the system size. The transition is no longer sharp but smooth and takes place over a finite temperature range^33^. The melting point corresponding to the temperature value is determined by monitoring the change where there is a jump in the slope of the caloric curves. The sudden increase in energy over a small temperature change indicates that the first order transition from solid to liquid phase is similar to previous work^34^. Figure 4a shows the caloric curves calculated from the NP1, NP2, NP3 and NP4. The NP1 shows a well-defined melting transition upon heating in the caloric curve. The change in energy of NP1 as the temperature increases is similar to that of monometallic NPs. For the NP2, NP3 and NP4, there is an irregular phenomenon of the melting that the energy decreases with the increase of the temperature. This is because that the Au atoms have lower surface energy, and the distribution of Au atoms on the surface layer can make the NPs in the state of low free energy. The sudden increase of the energy indicates that the total NPs melt. For NP5-NP8 in Fig. 4b, the irregular phenomenon occurs twice at least in the melting process. When the number of Au atoms increases, the Au atomic segregation leads to the decrease in the potential energy. After staying in a certain temperature, Au atoms will melt and release latent heat. When the energy increase in the temperature is more than the energy decrease induced by the atomic segregation, the first jump occur. At this time Ni NPs has not melted yet, Au atoms continue to wrap Ni NPs, repeating the energy reduction process until Ni melts, then occurring a second jump. There are at least one or two transition points on the potential energy curves due to the existence of a competition between the decreased energy induced by the atomic segregation and the increased energy as the temperature increases.

**Figure 5:**
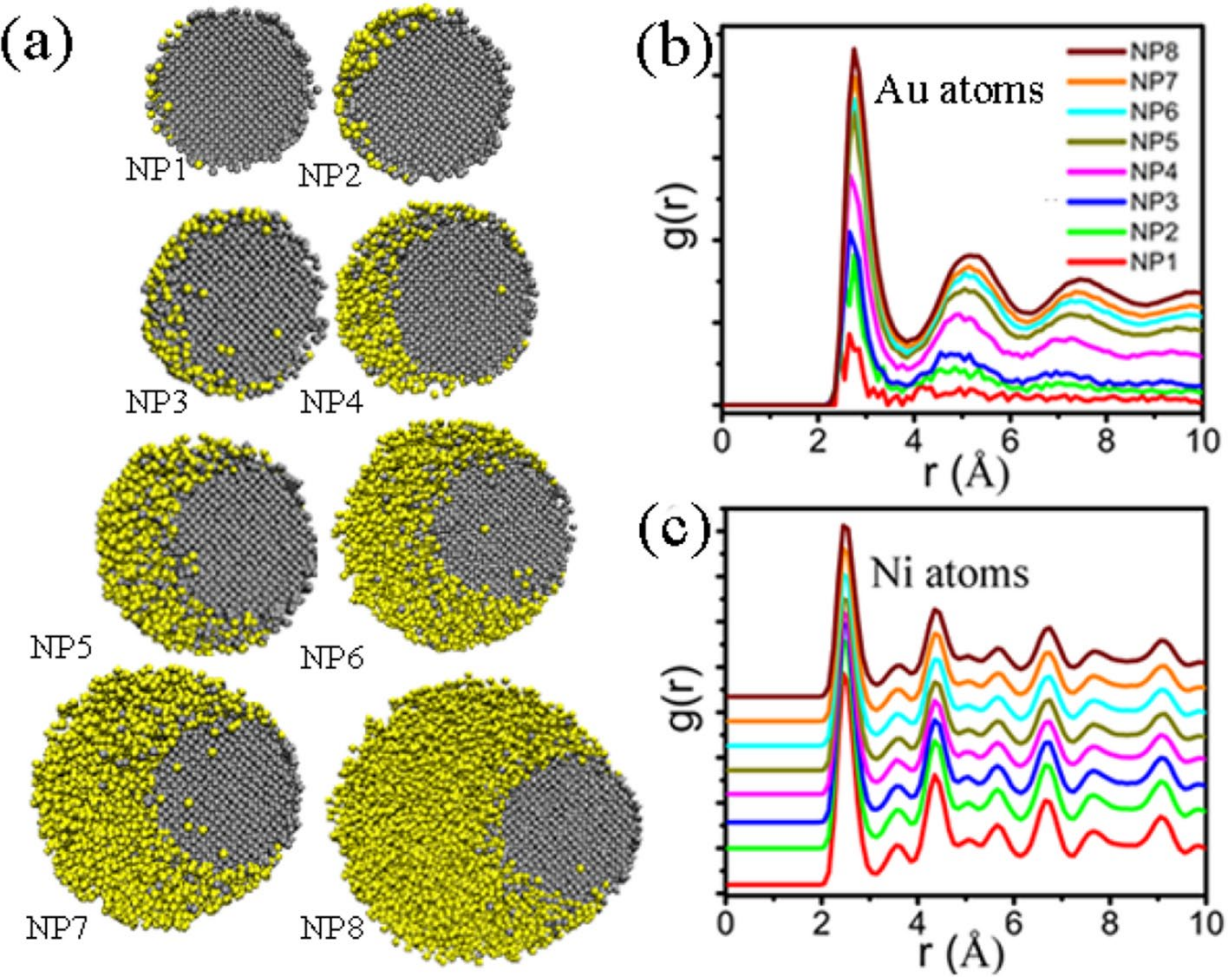
(**a**) Cross sections through the Au-Ni NPs at temperature 1173 K. (**b**,**c**) PDF of Au atoms and Ni atoms at temperatures 1173 K, Au and Ni atoms are colored in yellow and gray respectively.

To better understand the nature of the energy change, we select the cross section of the particle at temperature 1173 K in detail. As shown in Fig. 5a, Au atoms segregated to the surface of Ni particle is dominant during the continuous heating of NPs. The Au particles have melted and Ni particles are not melted. For NP1, NP2, NP3, Au atoms diffused along the surface of Ni. For NP4, NP5, NP6, Au and Ni form alloying at the interface besides the diffusion. This implies that Au and Ni can form alloy NPs although they are not mismatched in the bulk^35,36^. When Au is large enough (NP7, NP8), Au atoms encapsulate completely due to Au atoms segregation. With the Au atoms “flowing”, we can get the eccentric core–shell structure Au@Ni NPs. As shown in Fig. 5b,c, the PDF peaks reflect the local order of structure. The first PDF peak location on the curve corresponds to the nearest interatomic distances and the second peak corresponds to the lattice parameter. The Au atomic PDF of structure obtained at 1173 K are shown in Fig. 5b, and the corresponding PDF at 0.1 K are shown in Fig. S1a. There is only the first nearest-neighbor peak relative to the PDF at 0.1 K, and the other primary peak of FCC structures disappear. This indicates that PDF of Au atoms displays the transition from ordered structure to amorphous structure. The Ni atomic PDFs at 1173 K and 0.1 K are shown in Fig. 5c and Fig. S1b. As shown in Fig. 5c, the second peak position on the curve corresponding to the Ni particle lattice parameter, is found to be 0.355–0.365 nm, which is more than that of 0.1 K (0.351 nm). On the other hand, the width of the peak becomes wider. All characteristic peaks still occur. This illustrates that the Ni particles has similar characteristics (FCC structure) at 1173 K and 0.1 K. These observations also suggest that diverse melting modes occur during the continuous heating of Au-Ni NPs.

**Figure 6:**
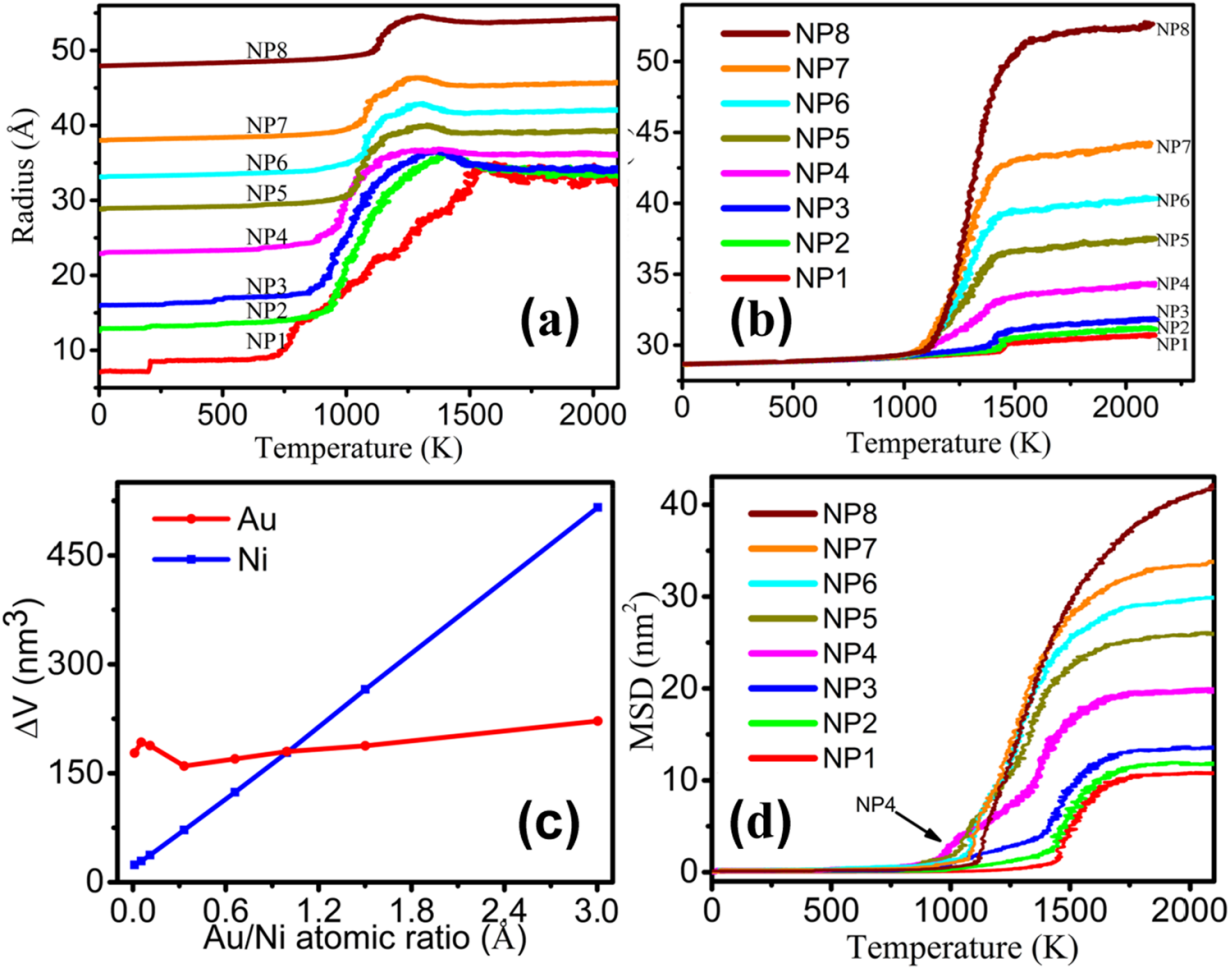
Evolution of radius of Au atoms (**a**) and Ni atoms (**b**) during heating process, (**c**) Volume change versus Au/Ni atomic ratio (**d**) MSD of NP1-NP8.

In order to obtain an intuitive size change of atomic diffusion in these Au-Ni NPs during the continuous heating, the concept of statistical radius was introduced into this work^37^. It can be seen from Fig. 6a,b that the statistical radii of Au and Ni NPs slowly increases with increasing temperature at low temperatures, which is usually attributed to the thermal expansion of lattice for the Au and Ni contents in the Au-Ni NPs. With the temperature further rising, the statistical radius abruptly increases with a bigger slope indicating the solid–liquid transition. Moreover, it can be noted from the curves that beyond the melting points, the statistical radii of Au are gradually decreased which is also caused by miscible between Au and Ni atoms. As shown in Fig. 6b, it can be noted that the statistical radii curves of Ni particles are obviously divided into two cases despite the same size of Ni particles. For NP1, NP2 and NP3, the radii of Ni particles become larger at high temperature, indicating that Ni particles require higher temperatures to deform. It is possible that the alloying only takes place on the surface because Au atoms only diffuse to the surface of Ni with a lower fraction of Au atoms in the NPs. Alloying between interior Ni atoms and Au atoms becomes almost impossible. For larger particle from NP4 to NP8, the transition is more apparent and occurs at low temperature. The reason could be that Au particles of larger size can simultaneously alloyed with Ni at the interface accompanied the diffusion process, which leads to the increase of the radius of Ni. To study the atomic arrangement of Au and Ni, volume increments of Ni and Au particles before and after melting are plotted in Fig. 6c. It can be seen that the ratio of Au and Ni atoms is 1:1, and the volume increment is the same. The reason could be that Au and Ni are miscible after melting. The mean square displacement (MSD) is a measure to describe the average distance that a particle travels in the sintering process. Figure 6d shows the MSD for the eight pairs of particles under the heating process. The MSD departs from the almost zero value and increases significantly, indicating the occurrence of atomic motion or melting. In general, when a small particle coalesces with a larger one, the temperature effect helps sintering where the smaller NPs tends to be incorporated into the larger ones^38^. The coalescence research of different sized gold NPs indicates that the melting occurs earlier when the size of the second particle is close to the first one^39^. The studies do not consider the weight of atoms due to the same element. However, as shown by the arrow in Fig. 6d, it is obvious that the atomic motion of Au-Ni NPs occurs earlier when the weights of Au and Ni particles are nearly the same according to our calculation (The NP4 being a typical representative). To perform an in-depth structural study, the MSD of Ni and Au atoms from NP1-NP8 are shown in the Fig. S2. The lighter particle tends to move towards heavier one. This illustrates that Au has a stronger affinity capacity relative to Ni when the size of Au particles is equal to that of Ni ones during the coalescence processes. The MSD of Ni and Au atoms almost experience a sharp rise simultaneously with the temperature increase, as can be seen from Fig. S2(d). Our atomic-scale observations clearly reveal that the weight plays a significant role during the process and the coalescence is driven by the surface diffusion and interface alloying. It can also be seen from Fig. S2 that the MSD from NP4 to NP8 provides direct evidence which indicates that Ni atoms are moved towards Au atoms.

**Figure 7:**
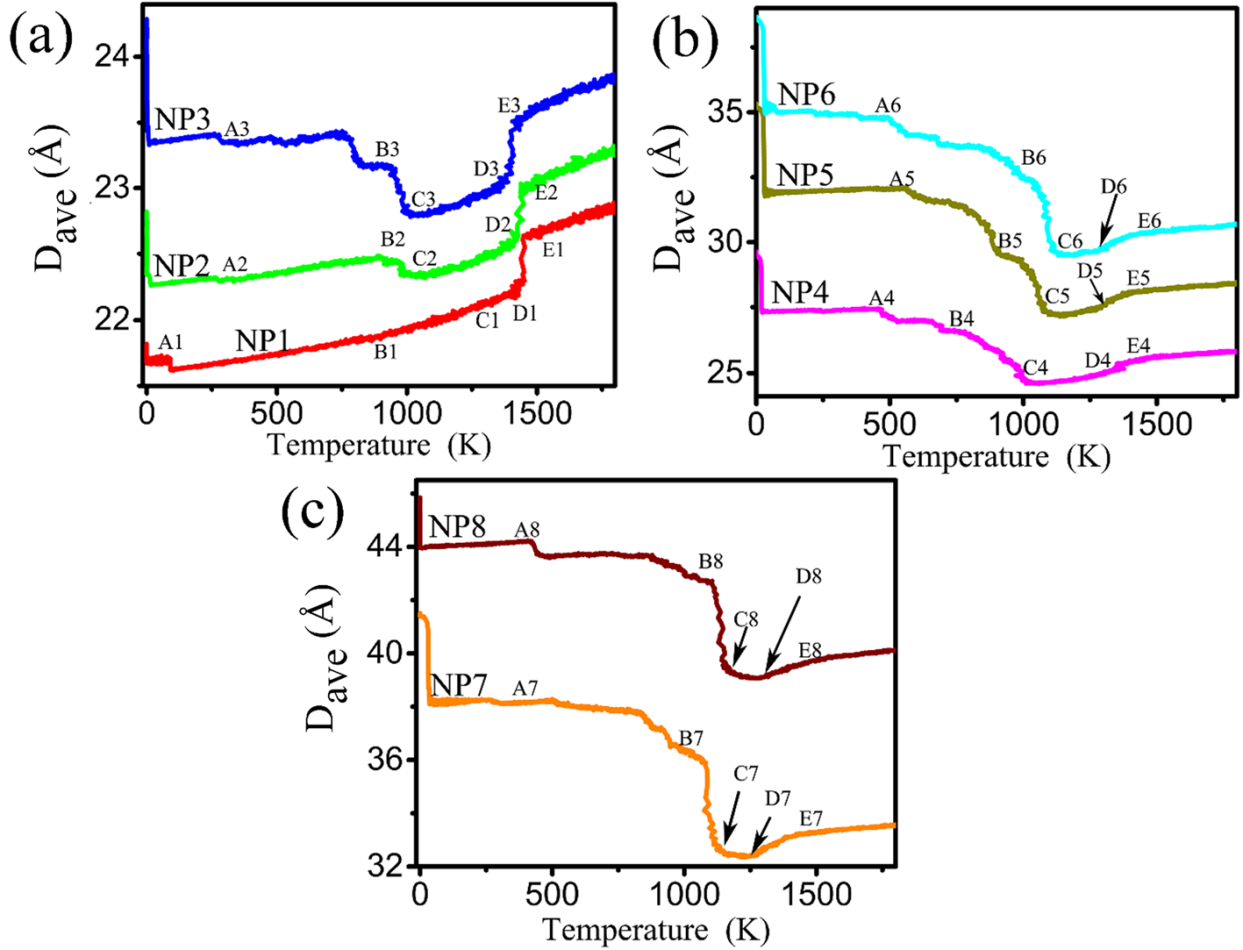
Temperature dependence of *D*_ave_, during the coalescence. (**a**) NP1, NP2, NP3 (**b**) NP4, NP5, NP6 (**c**) NP7, NP8.

To further investigate the change in morphology of the Au-Ni NPs in a quantitative way, the average distance *D*_ave_ between the Au/Ni atoms and the NPs center of coordinate is

$${D}_{ave}=\frac{1}{N}\sum_{1}^{N}\left|{r}_{i}-{r}_{C}\right|$$

where *r*_*i*_ is the position of the *i* atom and *r*_*C*_ is the center of coordinate. The temperature dependence of this amount was obtained by calculating *D*_ave_ at each temperature^12^. *D*_ave_ is sensitive to the shape of the Au-Ni NPs, and has a maximum value when the Au NPs and Ni NPs are separated and decreased when the NPs coalesce. A minimum value of *D*_ave_ is obtained for spherical Au-Ni NPs, which is the shape of some solid phase clusters or the liquid clusters. Here *D*_ave_ is employed to describe the coalescence process. According to the segregation of Au and the melting of Ni, the evaluation of *D*_*ave*_ of the NPs can be divided into three stages as shown in Fig. 7. Meanwhile, representative atomic morphology of the five representative temperatures of NP1-NP8 (in Table S2) is shown in Fig. S3. With respect to the behavior of the coalescence process, the dumbbell-like, Janus and eccentric core–shell spherical structure could be obtained during heating process.

*Dumbbell-like*: The first stage corresponds to the process from separate to contact state (Am points, m = 1–8) between Au particles and Ni particles, the sample changes are similar from NP1 to NP8, and the *D*_*ave*_ of NPs decreases sharply once the heating simulation starts. Then, the *D*_*ave*_ slightly increases or remains almost unchanged. This is because when the sintering neck was growing, the barycenter of system keep stable in the low temperature. The NPs are mainly in the form of dumbbell.

*Janus-like NPs*: After the coalesce process, the value of *D*_*ave*_ decreases as stage 2 (Am to Cm, m = 1–8). The *D*_*ave*_ of NP1 rapidly decreases at the point A1 due to the small number of Au atoms in NP1 and the rapid diffusion to the surface of Ni NPs. The *D*_*ave*_ of NP2 and NP3 decreases at point A2 and A3, and then slowly rises, but after that drops to the lowest point C2 and C3. As shown in the Fig. 7b, the *D*_*ave*_ of NP4, NP5 and NP6 continue to decrease from point A4, A5 and A6 to the lowest point C4, C5 and C6 respectively. This suggests that as the number of Au atoms increases, Au gradually diffuses to the Ni surface until a spherical structure is formed. And for NP7 and NP8, the most interesting phenomenon is that the *D*_*ave*_ decreases suddenly from B7 and B8 points to C7 and C8 points respectively. This indicates that the number of Au atoms is sufficient, and Ni NPs can be suddenly adsorbed into the Au NPs.

*The eccentric core shell NPs:* The stage 3 is corresponding to the melting process of Ni NPs (Dm points to Em points (m = 1–8) in Fig. 7.). The *D*_*ave*_ of NP1 to NP6 increases significantly with the rising temperature, which is corresponding to point C1, C2 ,C3, C4, C5 and C6 to point D1, D2, D3, D4 D5 and D6 respectively as shown in Fig. 7a,b. This is mainly due to the increasing temperature of the larger atomic spacing of Ni. In Fig. 7c, because *D*_*ave*_ does not reach the minimum points C7 and C8, the *D*_*ave*_ decreases slowly from points C7 and C8 points to D7 and D8 point respectively. This is due to the continuous wrapping process when Ni atoms are adsorbed inside Au particles. From Fig. S3, we can find that the NPs form a non-concentric structure for NP7 and NP8.

The morphology evolution in Fig. S3 conforms to the change of *D*_*av*e_ (maximum value means separate state, and minimum value represents the formation of a sphere). As can be seen in Fig. S3, Au atoms preferentially diffuse to the Ni surface^10,34^, so Au and Ni combine to form dumbbell, Janus and eccentric core shell structure. The in-situ TEM experimental results of structure evolution behaviors of NiAu nanospindles show that Au component wraps along Ni matrix to form a core–shell like structure at promoted temperature^16^. The conclusions directly verify our simulation results. The catalytic activity is highly dependent on the evolution of alloying and phase segregation^40,41^. The thermal control of the nanoscale alloying and phase segregation is of practical importance for its application as a catalyst and design of nanostructures.

## Conclusions

Using MD simulations, we have studied coalescence on Au-Ni NPs. Ni NPs has the same diameter of 5.8 nm and the Au NPs diameter ranged from 1.5 nm to 9.6 nm. We have investigated the contributions to the NPs coalescence process. Au atoms progressively diffuse to the surface of Ni NPs, which are in agreement with the existing TEM observations, and the alloying and segregation processes were found in these NPs. For small Au particles, the structure of sequence will change due to the lattice induction, and coalescence processes eliminate the interface defects of the NPs. With the increasing temperatures, change energy of small Au particles was similar as monometallic NPs, but for larger Au particles, the irregular variation of energy occurs and the atomic energy experiences at least one or two reductions. The weight ratio plays a primary role for relative motion between Au and Ni particles. In addition, this study indicates that the formation of various shapes of NPs, dumbbell, Janus and non-concentric spherical structure can be tuned by the coalescence temperature and Au content. When the number of Au atoms is sufficient, Ni NPs can be suddenly adsorbed into the Au NPs to form an eccentric core-shell spherical structure.

## Supplementary Information

Supplementary Information 1.

Supplementary Video 1.

Supplementary Video 2.
