# Supplementary material for: Molecular dynamics study on structural and atomic evolution between Au and Ni nanoparticles through coalescence
Source: Sci Rep. 2021 Jul 29;11:15432. doi: 10.1038/s41598-021-94822-0 (PMC8322430; doi:10.1038/s41598-021-94822-0)
Supplement: Supplementary file 1 — Supplementary Information 1. [file 41598_2021_94822_MOESM1_ESM.docx]

Molecular Dynamics Study on Structural and Atomic Evolution between Au and Ni Nanoparticles through Coalescence

Bangquan Li, ^1,2^ Jing Li^1^, Xiaoqiang Su^1^ and Yimin Cui^3^

^1^School of Physics and Electronics, Shanxi Datong University, Datong 037009, China

^2^Institute of Photoelectronic Thin Film Devices and Technology，Nankai University，

Tianjin 300350, China

^3^School of Physics, Beihang University, Beijing 100191, China

**Table S1** The illustration of the radius for different NPs

|  | NP1 | NP2 | NP3 | NP4 |
| --- | --- | --- | --- | --- |
| Au radius (nm) | **1.50** | **2.50** | **3.20** | **4.60** |
| Ni radius (nm) | **2.9** | | | |
| Atomic ratio | Au_0.01_Ni_0.99_ | Au_0.05_Ni_0.95_ | Au_0.1_Ni_0.9_ | Au_0.25_Ni_0.75_ |
|  | **NP5** | **NP6** | **NP7** | **NP8** |
| Au radius (nm) | **5.80** | **6.64** | **7.62** | **9.60** |
| Atomic ratio | Au_04_Ni_0.6_ | Au_0.5_Ni_0.5_ | Au_0.6_Ni_0.4_ | Au_0.75_Ni_0.25_ |
| Ni radius (nm) | **2.9** | | | |


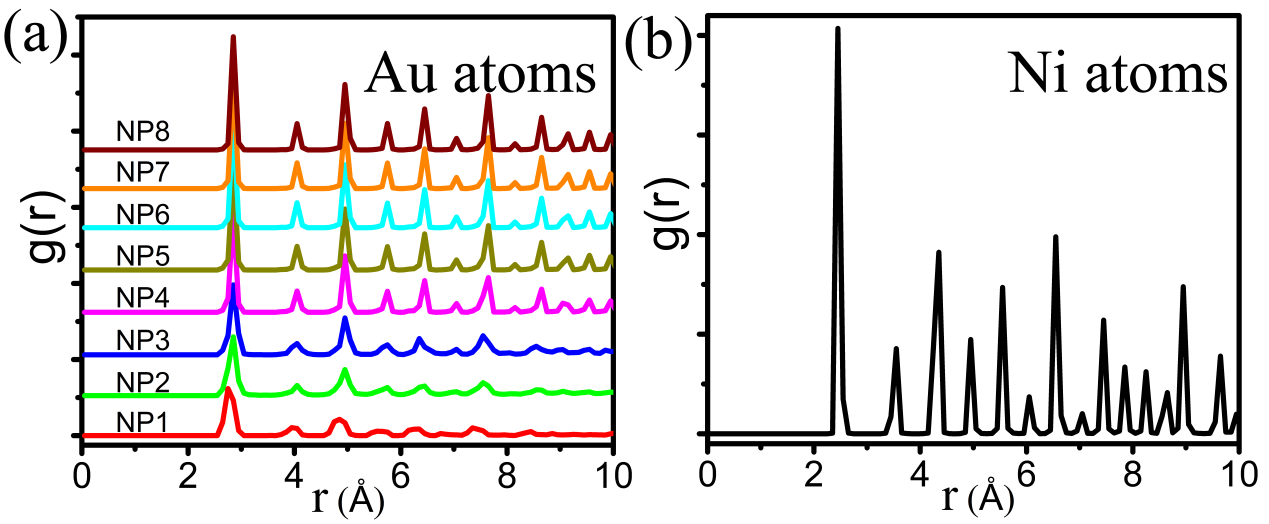


**Fig. S1** PDF of Au atoms and Ni atoms at temperatures 0.1 K

**Table S2** Five representative temperatures (K) of NP1-NP8 during the heating process

| Sample | T1 | T2 | T3 | T4 | T5 |
| --- | --- | --- | --- | --- | --- |
| NP1 | 97 | 960 | 1300 | 1401 | 1453 |
| NP2 | 288 | 960 | 1022 | 1423 | 1438 |
| NP3 | 300 | 922 | 1047 | 1380 | 1425 |
| NP4 | 464 | 712 | 1034 | 1340 | 1387 |
| NP5 | 558 | 907 | 1112 | 1271 | 1372 |
| NP6 | 477 | 874 | 1142 | 1281 | 1410 |
| NP7 | 247 | 1067 | 1142 | 1276 | 1452 |
| NP8 | 449 | 1087 | 1173 | 1319 | 1473 |


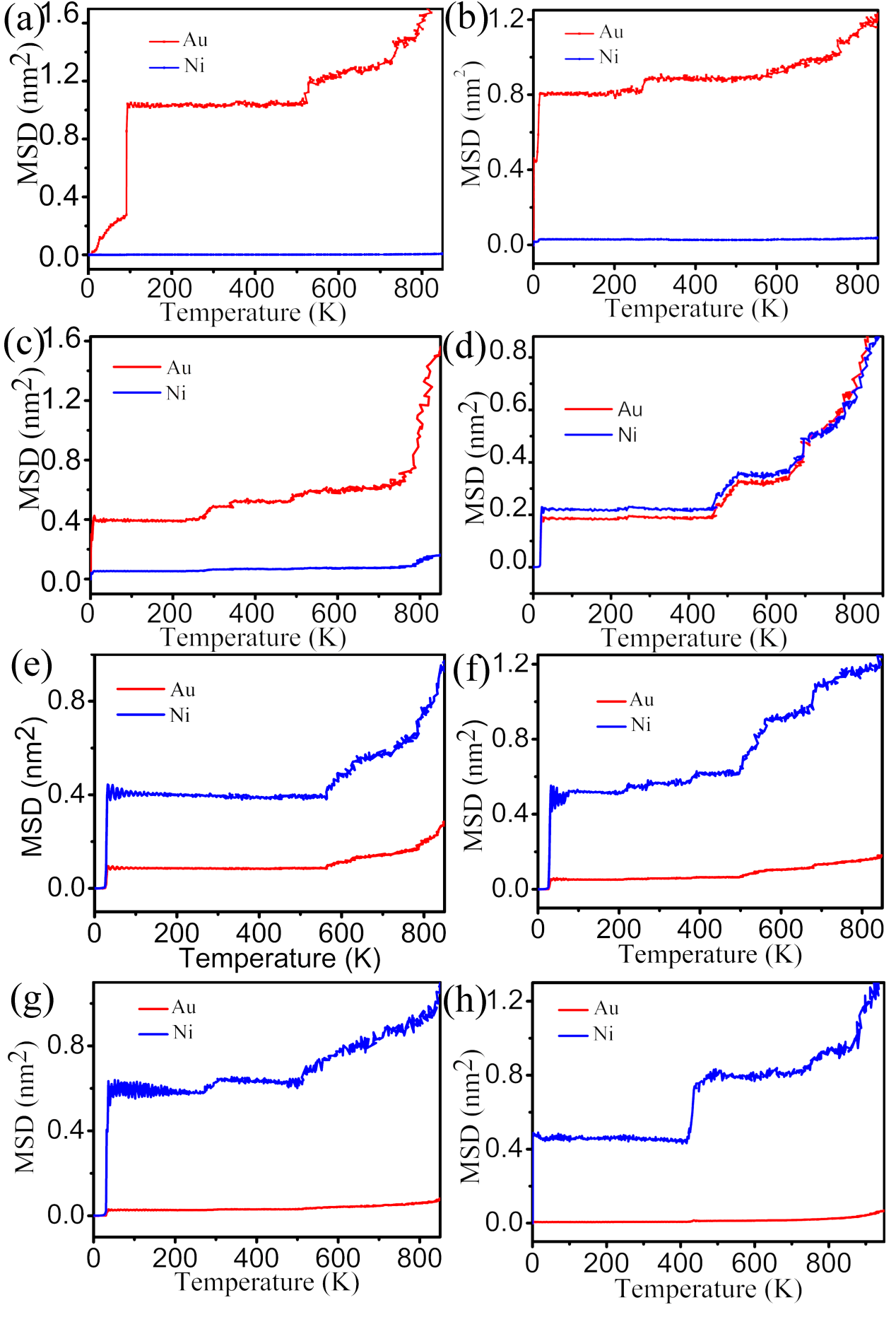


**Fig. S2** MSD of Ni and Au atoms in (a) NP1, (b) NP2, (c) NP3, (d) NP4, (e) NP5, (f) NP6, (g) NP7, (h) NP8 during heating process.


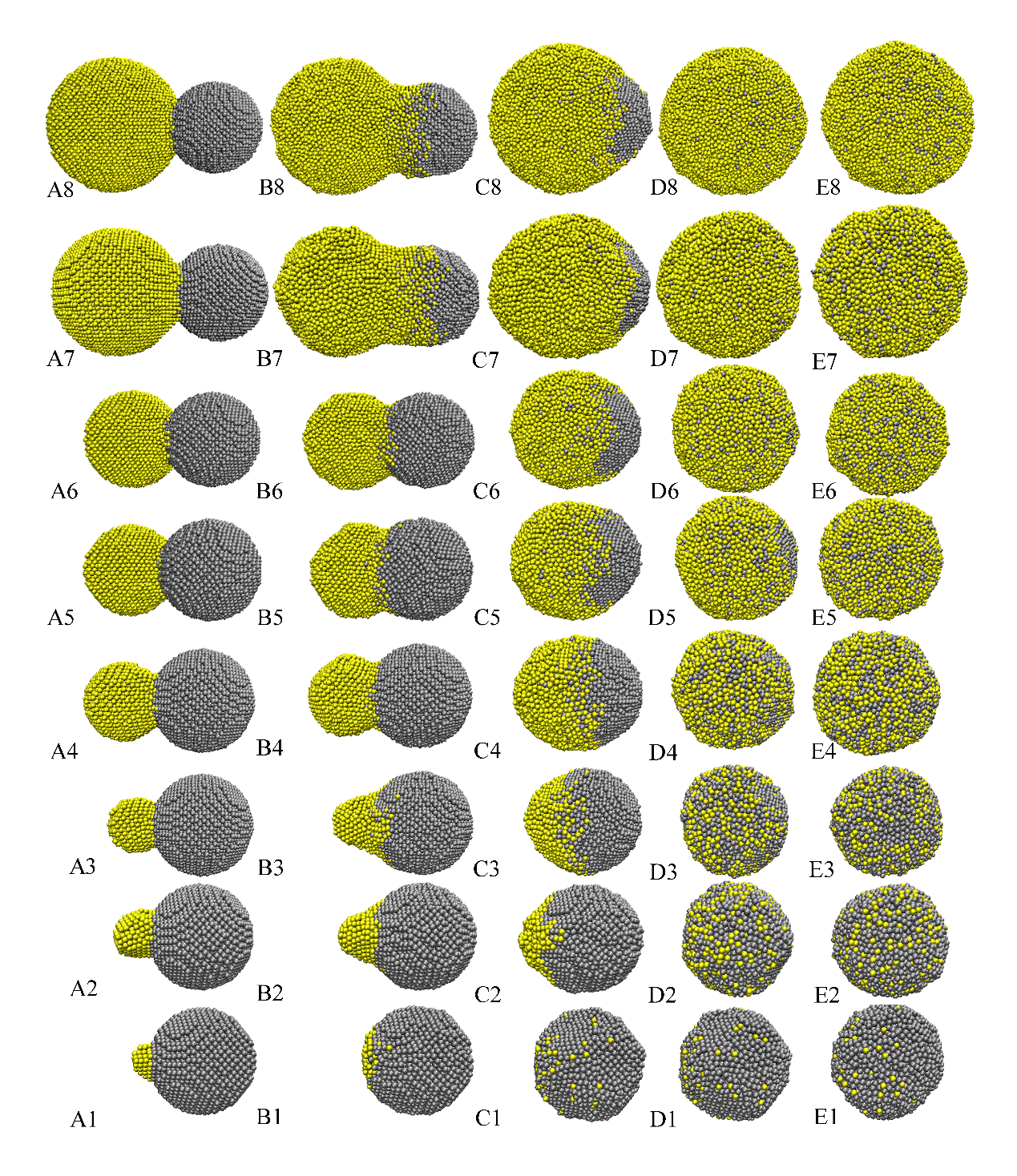


**Fig. S3** Snapshots at different temperatures during the coalescence, Au and Ni atoms are colored in yellow and gray, respectively.

Supplementary videos

**Video S1** Au particles undergo the shape convulsion and then coalesce with the large Ni particles in NP1.

**Video S2** The removal process of interface defect in NP1.
